# Ultrasonic liquid crystal tunable light diffuser

**DOI:** 10.1038/s41598-024-66413-2

**Published:** 2024-07-04

**Authors:** Yuma Kuroda, Ryoya Mizuno, Daisuke Koyama

**Affiliations:** Faculty of Science and Engineering, Doshisha University, 1-3 Miyakodani, Tatara, Kyotanabe, Kyoto 610-0321 Japan

**Keywords:** Ultrasound, Liquid crystal, Optical device, Vibration mode, Light diffuser, Electrical and electronic engineering, Adaptive optics, Mechanical engineering

## Abstract

Conventional light diffusers have periodic surface profiles, periodic refractive index distributions, or light scattering layers containing colloids. In all such structures the optical directivity of the light diffuser is cannot typically be controlled. Here we propose an electrically tunable light diffuser based on the application of ultrasound to a nematic liquid crystal (LC) material. The ultrasonic LC diffuser consists of an LC layer sandwiched by two glass discs and an ultrasonic transducer. The electrodes of the transducer are divided in a circumferential direction so that a resonant non-coaxial flexural vibration mode can be generated on the diffuser by controlling the electrical input signals. A continuous reversed-phase sinusoidal electric signal to the transducer generates the non-coaxial resonant flexural vibration mode on the glass disc, inducing an acoustic radiation force acting on the boundary between the LC layer and glass discs. This effect changes the molecular orientation of the LC and the transmitted light distribution. The diffusion angle of the transmitted light depends on the input voltage amplitude, and the diffusion angle was maximized at 16.0 V. The vibrational distribution and the diffusion directivity could be rotated by adjusting the input voltages to different electrodes, meaning that an ultrasonic LC diffuser with a thin structure and no moving mechanical parts provided a tunable light-diffusing functionality with rotatable directivity.

## Introduction

Light is essential to human life. Since the era when fire was used as a light source at night, humans have innovated various artificial light sources (e.g., incandescent lamps, gaslights, discharge lamps, and light-emitting diodes [LEDs])^1^. Indoor light intensity distribution is an important factor that affects humans’ ability to study and work efficiently^2^ as well as their physical and mental health^3^, based on how humans perceive light through stimulation of retina cells^4^. On this basis, modern artificial light sources are designed with these psychological elements in mind to achieve optimal aesthetics^5^. The high-energy efficiency of LEDs has contributed to the development of environmentally friendly and sustainable lighting systems; however, in the case of LEDs the light-emitting components tend to be smaller than legacy light sources, creating a challenge for illuminating wide spaces^6^. Hence, additional optical components, such as lenses, reflective plates, and diffusers are required to achieve a suitable light intensity distribution at low input energies. Thus, methods for controlling light directivity are of considerable practical importance in daily life.

Light refracts at the boundary between media with different refractive indices, such as between air and the surface of an optical lens, or a medium in which the refractive index has a spatial distribution. These two refraction behaviors can be considered by essentially the same approach^7^. The refractive angle depends on the refractive indices of the media and the incident angle (Snell’s law), meaning that the surface profile and positional relationship of the optical components determine the optical path of the transmitted light. To control the transmitted light, mechanical moving parts that can move or rotate the optical components are required; however, this is problematic in terms of reducing the overall size of the device. When the spatial distribution of the refractive index of optical components is non-uniform, incident light refracts gradually, even at a flat surface. Light propagates on the path with the shortest propagation time (Ferma’s law)^8^. Notably, graded index multimode fibers use this physical phenomenon to enable broadband optical communication^9^.

Conventional light diffusers have periodic surface profiles, periodic refractive index distributions, or light scattering layers with colloids in polymer layers^10^. Light diffusers with periodic surface profiles are fabricated through optical nanofabrication techniques such as photolithography^11^ and nanosecond laser-induced surface texturing^12^. In the case of diffusers with light scattering layers, colloidal materials such as titanium dioxide^13^ and silica dioxide^14^ are sealed in materials that have both high optical transparency and easy processability, such as paraffin^15^ and polystyrene^16^. Although the optical characteristics of these diffusers can be tailored in the fabrication process, the diffusion directivity cannot typically be changed after packaging. Light diffusors that enable control over the diffusion directivity could reduce energy consumption and achieve the required luminance intensity, enabling users to control luminance distribution after installation to provide more satisfying aesthetic results.

Sato developed a tunable optical lens with an inhomogeneous LC layer based on a change in the molecular orientation of the LC by application of an electric field toward the LC layer via transparent electrodes^17^. His group also improved the optical characteristic of this LC lens with the use of holed electrodes^18^. Several researchers have proposed and developed methods for controlling the optical path of transmitted light based on control of LC molecular orientation and electrically tunable light diffusers without mechanical moving parts. Khan et al*.* reported control of LC molecular orientation with the use of a spatially periodic electric field via transparent electrodes and monolayer of carbon nanotubes with polymer thin films, which generated periodic refractive-index distributions^19,20^. Zhou et al*.* used randomly oriented LC droplets wrapped in polymer thin films, where the LC molecular orientation could be changed by applying an electric field, to control the transmitted light diffusion^21,22^; however, the former approach required complex structures to generate a non-uniform electric field, and the diffusion directivity in the latter could not be controlled. The authors developed a technique to control the molecular orientation of LCs using ultrasonic vibration^23^ (rather than electric fields) and applied this technique to tunable lenses^24^. The lens was composed of an LC layer between two glass discs and a piezoelectric transducer. The input electrical signal to the transducer generated coaxial resonant flexural vibration modes on the lens that changed the orientation of the LC molecules and the spatial distribution of the refractive index^25,26^, resulting in convergence or divergence of the transmitted light. The focusing characteristics of the lens depended on the LC layer thickness and its birefringence^27^, and substrates with a hole the same size as the vibrational nodal circle of the lens were preferable for vibrational isolation^28^. The effective lens aperture could be controlled by adjusting the input voltage to the transducers^29^. However, to the best of our knowledge, an ultrasonically controllable optical diffuser based on LC materials has yet to be reported. In this paper, we propose a diffusing- and directivity-tunable ultrasonic LC light diffuser based on the generation of nonaxial resonant flexural vibration on the LC layer, which controls the molecular orientation and refractive-index distribution. The diffuser has a thin and simple structure with no mechanical moving parts and no complex-patterned transparent electrodes owing to the use of a piezoelectric transducer as the driving source.

## Methods

**Figure. 1:**
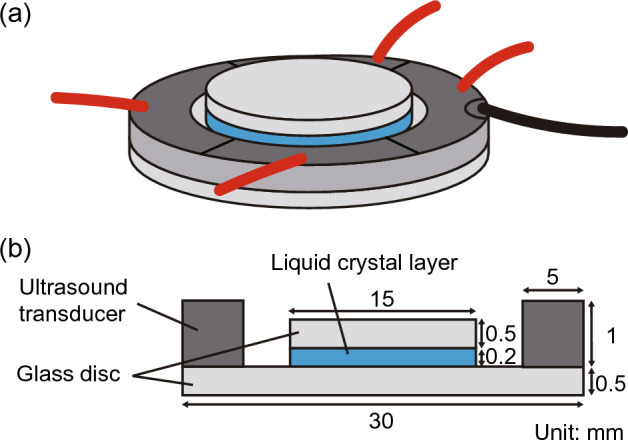
(**a**) Configuration and (**b**) cross-sectional view of the ultrasonic LC variable light diffuser.

**Figure. 2:**
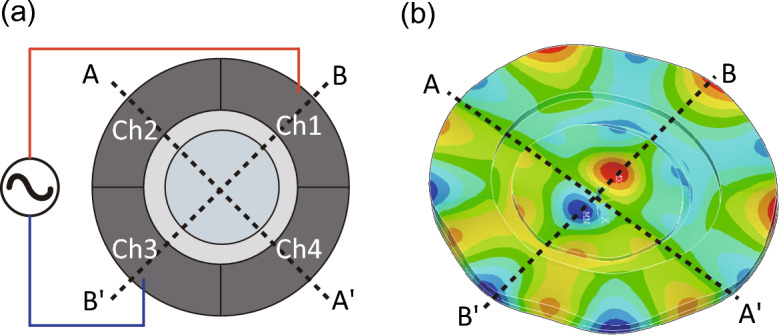
(**a**) Driving conditions and (**b**) vibration mode of the light diffuser calculated by FEA when a reversed-phase voltage of 64.5 kHz was applied to channels 1 and 3. The figure shows the instantaneous deformation; the red and blue parts represent the vibrational loops, and the green parts represent the vibrational nodes.

Figure 1 shows the configuration of the ultrasonic LC light diffuser. Two glass discs (diameter: 15 and 30 mm; thickness: 0.5 mm) with polyimide orientational films (SE-5611, vertically aligned type, Nissan Chemical, Tokyo, Japan) were fixed using epoxy via dimethylpolysiloxane spacers with a thickness of 0.2 mm to fabricate the LC layer. Nematic LC (4-cyano-4’-pentylbiphenyl, 5CB) was injected into the small gap between the discs by capillary action at atmospheric pressure and not under vacuum, and the surrounding part was sealed using epoxy. The orientational films were formed on the inner surfaces of the glass discs without rubbing, resulting in the vertical alignment of the LC molecules in the absence of ultrasound excitation. The authors have previously confirmed that a 0.2 mm-thick LC layer induces the largest change in the optical path of transmitted light as a tunable lens^27^. An annular piezoelectric transducer (inner diameter: 20 mm; outer diameter: 30 mm; thickness: 1 mm; lead zirconate titanate, C-213, Fuji Ceramics, Fujinomiya, Japan) polarized in the thickness direction was coaxially and parallelly attached to the larger glass disc using epoxy. The outer diameter of the transducer corresponded to the diameter of the larger glass disc. The surface electrode on one side of the transducer was divided into four in the circumferential direction; we refer to these separate electrodes as channels 1 to 4 in the anticlockwise direction (see Fig. 2a). The electrode on the attached side of the glass disc was the common ground electrode.

**Figure. 3:**
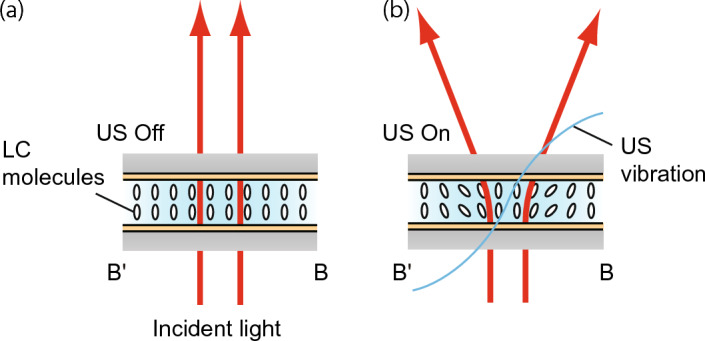
Schematics of the LC molecular orientation and the transmitted light (**a**) in the absence and (**b**) in the presence of the ultrasound excitation. Red and blue curves represent the light and the ultrasound vibration, respectively.

Ultrasound vibration is generated on the glass discs through the inverse piezoelectric effect on the application of a continuous sinusoidal electric signal to the transducer. When the driving frequency corresponds to the mechanical resonance frequency of the LC light diffuser, resonant flexural vibration modes are generated on the light diffuser at several frequencies. The flexural vibration on the glass discs generates differences in the acoustic energy density between the LC layer, the glass discs, and the surrounding medium (air) because of differences in the acoustic impedance. This effect induces an acoustic radiation force, that is a static ultrasonic force^30^, which changes the LC molecular orientation. Figure 2b shows the flexural vibrational mode of the LC light diffuser calculated by finite element analysis (FEA) using commercial FEA software (ANSYS 14.5, ANSYS, Inc., Canonsburg, PA, USA). Although there were several resonant frequencies of the light diffuser between 20 to 100 kHz, we used only one of the noncoaxial resonant flexural vibration modes generated at 64.5 kHz with one nodal line (line A–Aʹ in Fig. 2) by applying a reversed-phase continuous sinusoidal signal to two facing channels (channels 1 and 3). This effect is attributed to the vibrational distribution on the LC layer and the LC molecular orientation being correlated under ultrasonic excitation^26^. Figure 3 depicts a schematic of the light diffusion. The light diffusion is expected to be straight and the vibrational nodes and loops on the line B–Bʹ change the LC molecular orientation along the line through the acoustic radiation force, resulting in light refraction along the same direction. This means that the diffusion directivity and angle can be controlled by adjusting the input voltages to each channel. Additionally, the LC molecules used here are ellipsoid and have optical uniaxial anisotropy (the refractive indices of the LC molecule in the long and short axes are 1.53 and 1.72, respectively^31^) meaning that the transmitted light distribution of the light diffuser depends on the polarization direction of the incident light. In addition, the LC layer thickness is crucial to the optical characteristics of the light diffuser. The larger the LC layer thickness, the greater the phase retardation. If in-phase continuous sinusoidal signals are input to all the channels, axisymmetric resonant flexural vibration modes with a vibrational loop at the center and concentric vibrational nodal circles are generated. These modes exhibit an axisymmetric inclination of the LC molecular orientation from the center of the glass disc to the outer side in the radial direction because the acoustic radiation force acts from the vibrational loop to the nodes. This LC orientation can be utilized as a tunable optical lens^25^.

**Figure 4:**
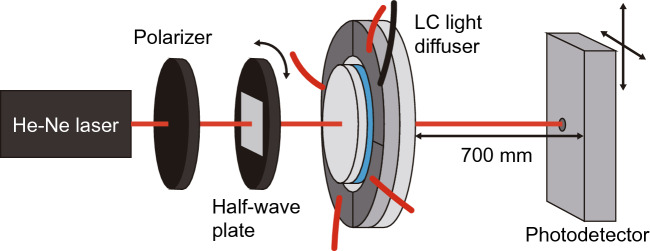
Experimental setup for optical measurements.

Continuous reversed-phase sinusoidal signals were input to channels 1 and 3 on the LC light diffuser using a function generator and a high-speed bipolar amplifier to generate the resonant flexural vibration mode, as shown in Fig. 2b. The out-of-plane vibrational distribution of the glass disc was measured using a laser Doppler vibrometer (LDV, VIO-130, Polytec, Waldbronn, Germany). A He-Ne laser beam (wavelength: 632.8 nm; output: 1 mW; full width at half intensity: 1.3 mm) was set to be perpendicularly incident to the center of the LC light diffuser via a polarizer and a crystal half-wave plate, and the transmitted light distribution was measured using a photodetector (sensor diameter: 0.9 mm, 2051, Newport, MA, US) with a pinhole (diameter: 2 mm) and a digital oscilloscope (see Fig. 4). The polarization direction of the incident beam was altered by rotating the half-wave plate. The vertical direction was defined as 0° polarization, and the counterclockwise direction was defined as the positive direction when viewed from the incident side. A vertical plane with an area of 10 × 10 mm^2^ at 700 mm from the light diffuser was scanned by the photodetector. The light diffuser was placed vertically, and the divided lines of the transducer’s electrodes correspond with the vertical and horizontal directions. Note that the change in the maximum light intensity with and without the light diffuser was within 5%. The change in the full width at half intensity of the beam was smaller than the spatial resolution of the measurements (0.5 mm). The refractive index distribution of the LC light diffuser in the absence of ultrasound was negligible and is therefore ignored. The two-dimensional refractive index distribution in the presence of ultrasound was observed using a birefringence profiler^32^, which converts the sample’s birefringence distribution into a first refraction intensity distribution using a digital camera module and diffraction gratings that exhibit specific polarization dependent. This process was explained in greater detail in our previous research^33^. The central region of the LC light diffuser, with an area of 6.7 × 6.7 mm^2^, was observed.

## Results and discussions

**Figure 5:**
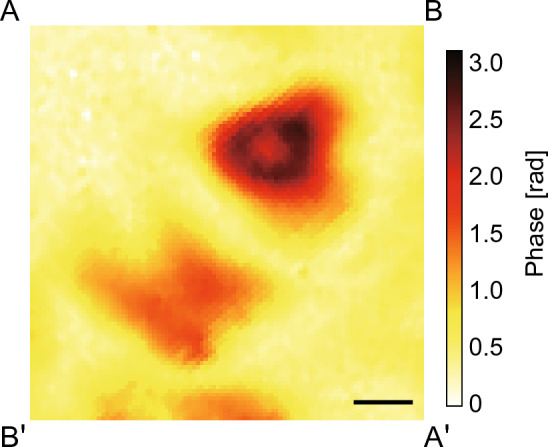
Two-dimensional phase difference distribution of the transmitted light through the LC light diffuser excited with 8 V at 65 kHz measured by the birefringence profiler. The scale bar indicates 1 mm.

**Figure 6:**
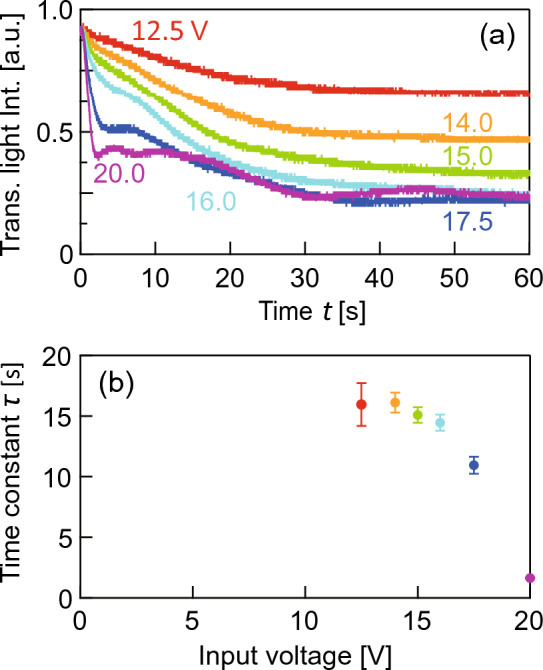
(**a**) Time response and (**b**) time constants of the transmitted light intensity at several input voltages.

**Figure 7:**
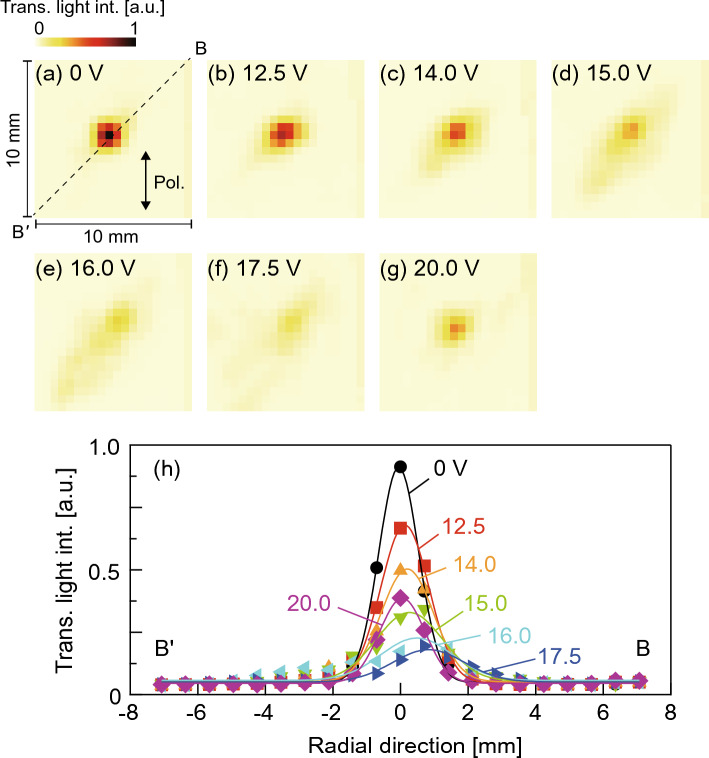
(**a**)–(**g**) Two-dimensional transmitted light intensity distributions and (**h**) the distributions on line B–Bʹ (dotted line in (**a**)) when changing the voltage amplitude of reversed-phase signals to channels 1 and 3. Arrow in (**a**) indicates the polarization direction of the incident light of 0°.

There were several resonant frequencies on the LC light diffuser between 20 to 200 kHz (see Supplement 1, Fig. S1). The resonant flexural vibration mode, as shown in Fig. 2b, was generated on the prototype at 65 kHz. In this paper, we used this vibration mode to investigate the diffusion characteristics. Figure 5 shows the two-dimensional phase difference distribution of the transmitted light in the center region of the LC light diffuser measured by the birefringence profiler in the case that continuous reversed-phase sinusoidal signals were applied to channels 1 and 3 (see Supplementary Movie). Two peaks in the refractive index were observed on the line B–Bʹ, as illustrated in Fig. 3b. Figure 6a shows the changes in the transmitted light intensity of the LC light diffuser with respect to time in the cases with different voltage amplitudes. At *t* = 0, channels 1 and 3 began receiving continuous reversed-phase sinusoidal signals. The transmitted light intensities on the vertical axis were normalized against the initial value at *t* = 0. The transmitted light intensities decreased gradually upon the onset of ultrasound excitation, and the terminal value decreased with the input voltage, meaning that the transmitted light was scattered away from the optical axis and into the surrounding areas. Figure 6b shows the relationship between the time constant *τ* and the input voltage amplitude when the polarization direction of the incident light was 0°. The time constant was calculated by assuming that the response curves of the transmitted light intensity can be expressed as an exponential function: *I*_0_ exp(− *t*/*τ*) + *I*_1_, where *I*_0_ is the constant, *I*_1_ is the terminal value at *t* = 60 s, and *I*_0_ + *I*_1_ = 1. The error bars were calculated from the sum of the background white noise generated by the photodetector and oscilloscope (≈20 mV), and the plots indicate their median values. Figure 7a to g show the two-dimensional transmitted light intensity distributions when changing the input voltage from 0 to 20 V. The light intensities from each condition were normalized against the maximum value at 0 V, and the polarization direction of the incident light and line B–B´ are indicated. Figure 7h shows the transmitted light distributions on the line B–Bʹ. The transmitted light intensity became lower at the center and diffused in the B–Bʹ direction as input voltage was increased, indicating that the diffusing angle of the transmitted light can be controlled via input signal voltage. Notably, the light intensity at the center increased again at 20 V.

As shown in Fig. 6b, the time constants of the diffusion response changed little when the input voltage was less than 17.5 V, indicating that the acoustic radiation force acting on the LC molecules and the elastic restoring force of the LC molecules were balanced in a steady state. The response times of typical LC optical devices were proportional to the square of the LC layer thickness^34^ and the diffusion angle increases as the LC layer thickness increases because the transmitted light follows Frema’s law, and the optical path length is the dominant factor. Hence, there is a trade-off between the response time and the diffusion angle. However, it is important to note that an increased LC thickness may pose a risk of inducing dynamic scattering^25^ at low voltage, due to a reduction in the anchoring force of the orientational films to the LC molecules in the center region in the thickness direction^27^. In addition, it is possible that the optical characteristics may be affected by the gravitational forces acting on the LC molecules (see Supplement 1, Fig. S2). Dynamic scattering occurs in LC devices using electric fields^35^, and this unstable phenomenon is attributed to turbulence in the LC layer induced by external forces, resulting in the light scattering in the LC layer and the fluctuation of the transmitted light. Dynamic scattering was observed in our ultrasonic LC devicess^25^.When the input voltage exceeded 17.5 V, the response curve shape became unstable (see Fig. 6a). This behavior is attributed to dynamic scattering of the LC molecules generated by the high-intensity ultrasound. In the absence of ultrasonic excitation, the intermolecular force, anchoring force associated with the orientation of the polyimide films, and thermal diffusion flow acting on the LC molecules^36^ are balanced, such that the LC molecules align vertically to the glass discs. On the application of the ultrasound, the acoustic radiation force generated by the ultrasonic vibration acts on the LC molecules^26^, resulting in inclination of the LC molecules from their initial state. If the acoustic radiation force is comparatively small, the LC molecules begin to rotate and reach a balanced angle. However, if the acoustic radiation force exceeds a threshold, the LC molecules rotate and vibrate unstably, breaking the mechanical equilibrium. This hypothesis is supported by the fact that the response curves measured at 17.5 V and above had a short time constant but were unstable; hence, the LC light diffuser should be used below this excitation threshold in real-world applications. The increase in the light intensity observed at the center at 20.0 V can be attributed to the fact that the incident light was not diffused effectively due to the generation of dynamic scattering (see Fig. 7h). Note that the response of the ultrasound LC device was highly reproduceable, i.e., the confidence interval of the change in the optical path was within ±1.8% of the mean value at a 99% confidence level with 30 measurements below the threshold voltage^37^. The maximum diffusion angle was calculated as 0.24° from the half width at half intensity when the input voltage was 16.0 V (in the absence of ultrasound excitation, the diffusion angle was 0.08°). The maximum diffusion angle is dependent on the wavelength of incident light and the birefringence of the LC material. A LC material with a greater birefringence will result in a larger diffusion angle, as the inclination of the LC molecules is the source of light diffusion. The diffusion angle of this LC light diffuser remains relatively constant between 400 and 800 nm because the refractive indices of 5CB we used remain stable within this wavelength band^38^.

**Figure 8:**
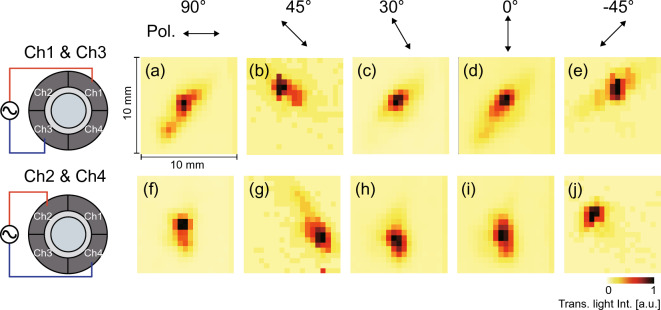
Transmitted light intensity distributions in the cases with several polarization directions of the incident light; (**a**) to (**e**) for channels 1 and 3 drive and (**f**) to (**j**) for channels 2 to 4 drive.

If channels 2 and 4 were used instead of channels 1 and 3 for the reversed-phase signal inputs of 15 V, the vibrational distribution on the LC light diffuser and the transmitted light intensity distribution could be rotated by approximately 90° (see Fig. 8). These results mean that the light diffusion directivity can be controlled by selecting a combination of input channels, and the rotation resolution may be improved by increasing the number of divided electrodes on the transducer, e.g., 8 or 12 divided transducers will realize 45° or 30° step resolution, respectively. The change in the rotation angle of the light diffusion did not correspond to 90° exactly, which is attributed to the fact that the LC device was fabricated by hand in our laboratory, which may have resulted in an inhomogeneous LC thickness and vibration. In fact, the frequency characteristics of the electrical admittance exhibited slight differences between channels 1 and 3 and channels 2 and 4. Furthermore, the injection of the LC under atmospheric pressure during the fabrication process would also result in a slight inhomogeneous vertical alignment in the default state. More precise and controllable methods for packaging liquid crystal materials (for example through microfluidic techniques^39^) should be used in the future.

The effects of the polarization direction of the incident light on the transmitted light intensity were investigated by rotating the half-wavelength plate and the polarization plane (see Fig. 8a to e). The diffusion angles in the B–Bʹ direction for the polarization directions of 90°, 0°, and − 45° were greater than that for 30°; the diffusion angle was dependent on the polarization direction of the incident light. This is because the LC molecular orientation changes in the perpendicular direction to the vibrational nodal line (B–Bʹ direction), and the diffusion angle is maximized when the polarization direction corresponds to this direction. Thus, the diffusion light pattern is determined by the vibration mode of the light diffuser, the polarization plane, and the beam width of the incident light.

## Conclusion

An electrically tunable light diffuser based on ultrasonic vibration and a nematic LC material is developed. The ultrasonic LC device has a simple and thin structure with no mechanical moving parts. The non-coaxial resonant flexural vibration mode generated by a reversed-phase drive was used for the light diffusion functions. The transmitted light through the LC light diffuser was scattered in one direction owing to the change in the LC molecular orientation induced by the acoustic radiation force. The transmitted light intensity distribution depended on the polarization direction of the incident light and the vibrational distribution of the light diffuser. By adjusting the driving signals to the divided electrodes of the ultrasound transducer, the diffraction angle and direction could be controlled.

### Supplementary Information

Supplementary Video 1.

Supplementary Information.

## Data Availability

The datasets generated during and/or analyzed during the current study are available from the corresponding author on reasonable request.

## References

[1] Guarnieri M (2018). An historical survey on light technologies. IEEE Access.

[2] Pimputkar S, Speck JS, DenBaars SP, Nakamura S (2009). Prospects for LED lighting. Nat. Photonics.

[3] Svilainis L (2007). LED directivity measurement in situ. Measurement.

[4] Cajochen C (2007). Alerting effects of light. Sleep Med. Rev..

[5] Boyce PR (2021). Light, lighting and human health. Light. Res. Technol..

[6] Int J (2008). Svilainis, L. LED directivity measurement in situ. Meas. Meas. Confed..

[7] Golomb M (1964). Elementary proofs for the equivalence of Fermat's principle and Snell's law. Am. Math. Mon..

[8] Born M, Wolf E (2013). Principles of Optics.

[9] Ishigure T, Nihei E, Koike Y (1994). Graded-index polymer optical fiber for high-speed data communication. Appl. Opt..

[10] Zhou L, Liu S, Zhong T (2022). A comprehensive review of optical diffusers: Progress and prospects. Nanoscale.

[11] Yamashita K, Kunitsu K, Hattori T, Kuwahara Y, Saito A (2021). Demonstration of a diffraction-based optical diffuser inspired by the Morpho butterfly. Opt. Express.

[12] Alqurashi T (2017). Femtosecond laser directed fabrication of optical diffusers. RSC Adv..

[13] Huang T, Ciou J, Huang P, Hsieh K, Yang S (2008). Fast fabrication of integrated surface-relief and particle-diffusing plastic diffuser by use of a hybrid extrusion roller embossing process. Opt. express.

[14] Zhong X (2017). Synthesis of organosiloxane-coated SiO_2_/CeO_2_ with multilayered hierarchical structure and its application in optical diffusers. J. Mater. Sci..

[15] Moagăr-Poladian G (2020). Paraffin as a material for optical diffusers – Fabrication and characterization. Opt. Mat..

[16] Wang M, Ye X, Wan X, Liu Y, Xie X (2015). Brilliant white polystyrene microsphere film as a diffuse back reflector for solar cells. Mater. Lett..

[17] Sato S (1979). Liquid-crystal lens-cells with variable focal length. Jpn. J. Appl. Phys..

[18] Ye M, Wang B, Sato S (2004). Liquid-crystal lens with a focal length that is variable in a wide range. Appl. Opt..

[19] Khan AA (2014). Tunable scattering from liquid crystal devices using carbon nanotubes network electrodes. Nanoscale.

[20] Butt H (2017). Electrically tunable scattering from devitrite-liquid crystal hybrid devices. Adv. Opt. Mater..

[21] Zhou L (2018). A novel light diffuser based on the combined morphology of polymer networks and polymer balls in a polymer dispersed liquid crystals film. RSC Adv..

[22] Ma H, Zhou L, Han C, Zhang C, Zhang L (2018). The fabrication of novel optical diffusers based on UV-cured polymer dispersed liquid crystals. Liq. Cryst..

[23] Taniguchi S (2016). Control of liquid crystal molecular orientation using ultrasound vibration. Appl. Phys. Lett..

[24] Shimizu Y (2018). Ultrasound liquid crystal lens. Appl. Phys. Lett..

[25] Harada Y (2020). Molecular orientation in a variable-focus liquid crystal lens induced by ultrasound vibration. Sci. Rep..

[26] Iwase T (2022). Orientation angles of liquid crystals via ultrasound vibrations. Jpn. J. Appl. Phys..

[27] Iwase T, Onaka J, Emoto A, Koyama D, Matsukawa M (2022). Relationship between liquid crystal layer thickness and variable-focusing characteristics of an ultrasound liquid crystal lens. Jpn. J. Appl. Phys..

[28] Kuroda Y (2023). How to fix an ultrasonic variable-focus liquid crystal lens for substrate-mountable applications. Jpn. J. Appl. Phys..

[29] Onaka J, Iwase T, Fukui M, Koyama D, Matsukawa M (2021). Ultrasound liquid crystal lens with enlarged aperture using traveling waves. Opt. Lett..

[30] Doinikov AA (1996). Theory of acoustic radiation pressure for actual fluids. Phys. Rev. E.

[31] Pizzey C, Van Duijneveldt J, Klein S (2004). Liquid crystal clay composites. Mol. Cryst. Liq. Cryst..

[32] Emoto, A., Otani, N. & Fukuda, T. US Patent 10119904 (2018).

[33] Onaka J, Koyama D, Kuroda Y, Emoto A, Matsukawa M (2022). Optical evaluation of a double-layered ultrasound liquid crystal lens. J. Appl. Phys..

[34] Jakeman E, Raynes EP (1972). Electro-optic response times in liquid crystals. Phys. Lett. A.

[35] Heilmeier GH, Zanoni LA, Barton LA (1968). Dynamic scattering: A new electrooptic effect in certain classes of nematic liquid crystals. Proc. IEEE..

[36] Khoo IC (2009). Nonlinear optics of liquid crystalline materials. Phys. Rep..

[37] Kuroda Y, Harada Y, Emoto A, Matsukawa M, Koyama D (2024). Frequency characteristics of an ultrasonic varifocal liquid crystal lens. Appl. Opt..

[38] Li J, Wu S (2004). Extended Cauchy equations for the refractive indices of liquid crystals. J. Appl. Phys..

[39] Whitesides GM (2006). The origins and the future of microfluidics. Nature.

