# Supplementary material for: Ultrasonic liquid crystal tunable light diffuser
Source: Sci Rep. 2024 Jul 4;14:15445. doi: 10.1038/s41598-024-66413-2 (PMC11224247; doi:10.1038/s41598-024-66413-2)
Supplement: Supplementary file 2 — Supplementary Information. [file 41598_2024_66413_MOESM2_ESM.docx]

**Ultrasonic liquid crystal tunable light diffuser: supplemental document**

# Frequency characteristics

There were several resonant frequencies on the LC light diffuser between 20 to 200 kHz. This paper focuses on the noncoaxial flexural vibration mode at 65 kHz, as this mode exhibited the largest vibrational displacement amplitude and change in the refractive index across all resonant frequencies. The refractive index of the LC light diffuser can be altered at the other resonant frequencies. Figure S1 shows the two-dimensional phase difference distribution of the transmitted light when the light diffuser was excited with 25 V at a resonant frequency of 34 kHz. The peaks of the phase difference on the line B–B´ were observed as in the experiment at 65 kHz.


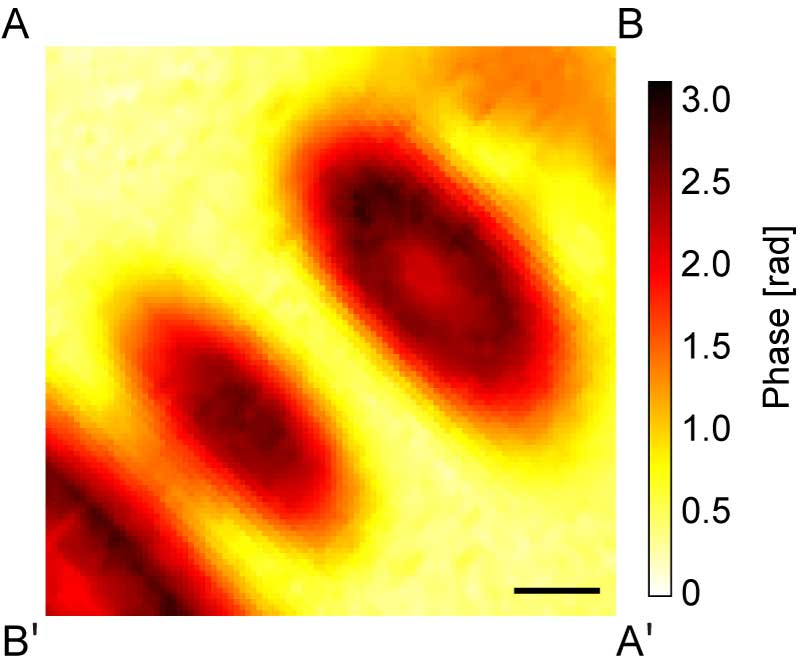


Figure. S1 Two-dimensional phase difference distribution of the transmitted light through the LC light diffuser excited with 25 V at 34 kHz measured by the birefringence profiler. The scale bar indicates 1 mm.

# Effects of gravity

The LC light diffuser is composed of a 200-μm-thick LC layer, which is relatively thick in comparison to the typical LC devices. It is therefore possible that the optical characteristics may be affected by the gravity. The optical images of the vertically and horizontally positioned diffuser were observed using a polarization microscope under the cross-Nicol condition to investigate the effect of gravity on the diffuser’s behavior. Figures S2a and b show the optical images of the center region of the vertically and horizontally positioned diffuser, respectively, in the absence of ultrasound excitation. These images suggest that the LC molecules are orientated vertically with minimal variation between them. This indicates that the gravitational forces acting on the device can be disregarded under the experimental conditions.


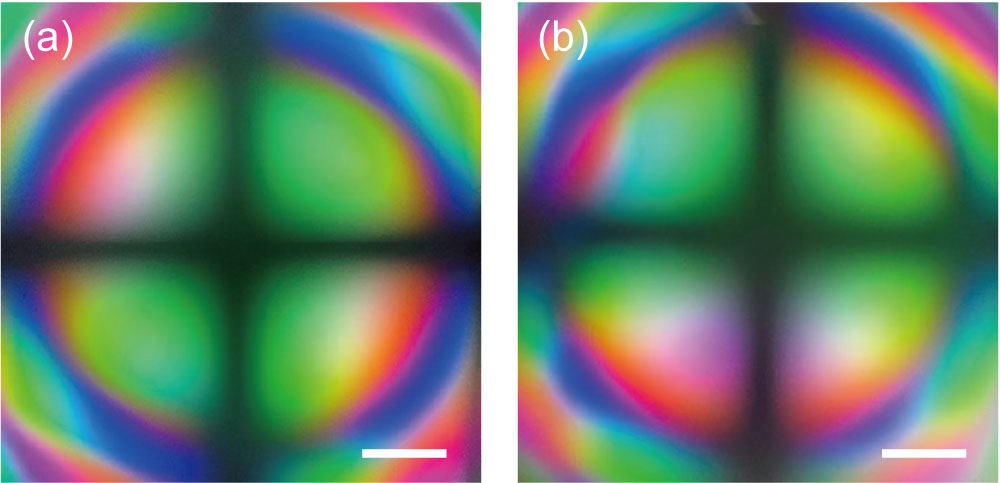


Figure. S2 Optical images of (a) the vertically and (b) horizontally positioned light diffuser observed by the polarization microscope. The scale bars indicate 3 mm.
